# Bacterial Microbiota Signatures Suggest Acclimation of the Gorgonian *Leptogorgia sarmentosa* to Highly Impacted Environments in the Mediterranean Sea

**DOI:** 10.1007/s00248-026-02809-z

**Published:** 2026-06-18

**Authors:** Elena Quintanilla, Janire Salazar, Jeroen van de Water, Teresa Madurell

**Affiliations:** 1 Department of Integrative Marine Ecology, Stazione Zoologica Anton Dohrn, Genoa Marine Centre, Genoa, Italy; 2 Institut de Ciències del Mar (ICM-CSIC), Barcelona, Spain; 3 Programa de Ciències del Mar, Facultat de Ciències de La Terra, Universitat de Biologia (UB), Barcelona, Spain; 4 Department of Estuarine and Delta Systems, Royal Netherlands Institute for Sea Research (NIOZ), Yerseke, The Netherlands

**Keywords:** Western mediterranean, Gorgonians, Coral microbiome, Microbiome flexibility, Harbour

## Abstract

Anthropogenic disturbances increasingly compromise marine environments, with severe consequences for vulnerable coral ecosystems. While global stressor impacts on stony corals are well-documented, the mechanisms by which local perturbations influence gorgonians remain poorly characterized. This limits our ability to predict the tolerance of temperate octocorals in human-dominated coastal landscapes. The Mediterranean gorgonian *Leptogorgia sarmentosa* is remarkably resilient, thriving in both marine protected areas (MPAs) and highly impacted urban habitats. To investigate the microbiota's role in this adaptability, we characterized the bacterial communities of *L. sarmentosa* from a protected site (Western Mediterranean) and an impacted seaport (Barcelona). For broader regional context, results were compared in parallel with a re-analysis of datasets from similarly contrasting Mediterranean sites (Cassis and La Spezia). Our results reveal distinct site-specific microbial signatures, but no differences in alpha diversity or dispersion between seaport and the MPA. However, significant compositional shifts occurred in the impacted Barcelona seaport, characterized by a reconfiguration of the dominant symbiont genus *Endozoicomonas* and an enrichment of *Spongiibacteraceae*_clade BD1-7. Similarly, *Endozoicomona*s strain abundances differed between Cassis and the high-runoff environment of La Spezia, which exhibited increased *Mycoplasma* abundance. Collectively, these findings suggest a high degree of microbiome flexibility. This microbiota plasticity, alongside predicted functional pathways, suggests a contribution to preserving and acquiring key holobiont functions, highlighting the capacity of *L. sarmentosa* to persist in disturbed habitats through strategic microbial reconfiguration. This study provides crucial insights into the mechanisms underlying coral acclimation; essential for developing conservation strategies and predicting the long-term viability of Mediterranean marine biodiversity.

**Supplementary Information:**

The online version contains supplementary material available at 10.1007/s00248-026-02809-z.

## Introduction

The orange sea fan*, Leptogorgia sarmentosa* (Esper, 1791), an azooxanthellate gorgonian, is one of the most frequent corals inhabiting the northwestern Mediterranean benthic assemblages [1–5]. With a broad depth distribution, ranging from shallow waters down to the continental shelf [5–7], it is commonly found in high densities on gravel bottoms, scattered across soft sediments, and on coralligenous substrates [4–6, 8]. This species is also typically associated with strong currents and turbid waters [1, 3, 9]. It is a fast-growing species [10], capable of thriving in harsh environmental conditions such as seaport habitats [3]and under high seawater temperatures [2, 11]. *L. sarmentosa* is therefore considered a tolerant and resilient gorgonian, showing greater resistance to stressors compared to other gorgonian species [12–14]

Given the vulnerable nature of coral ecosystems, understanding the mechanisms underlying the occurrence and persistence of coral species in highly perturbed environments is essential for distinguishing true adaptive strategies [15, 16]. It also aids in predicting which coral communities are more likely to recover after global and local-scale disturbances [17]. Thus, by identifying resilient species and uncovering the traits that support their survival and adaptation, we can recognize species that may act as ecological anchors or form refugia within collapsing reef systems [18, 19]. While the biological and physiological traits underlying gorgonians’ responses to environmental change have been relatively well studied [12, 20–22], the role of microbiome-associated traits and their contribution to resilience and acclimation remain comparatively underexplored.

Corals and gorgonians are considered holobionts, complex ‘meta-organisms’ and ecological units comprising the coral host and the associated microbial communities (i.e. the microbiota) including protists, bacteria, archaea, viruses and fungi [23, 24]. The coral-associated microbiota plays a critical role in coral holobiont health, contributing to nutrient cycling, protection against pathogens, and aiding in adaptation to environmental changes [25–27]. In corals, a concept known as ‘microbiome flexibility’ [28] was recently introduced, posing that some corals may strictly maintain the composition of their microbiota (‘regulators’), whereas others may modify their microbiota (‘conformers’) to facilitate rapid responses to environmental stressors. Therefore, understanding the microbiota composition and its dynamics may serve as a valuable indicator of environmental stress and a potential predictor of coral tolerance under disturbed conditions.

Microbiome studies on Mediterranean gorgonians are relatively recent. Initial exploratory work on the microbiota composition of Mediterranean gorgonians, indicated that *L. sarmentosa* has a flexible microbial community, which may allow for adaptation to local conditions [29–31]. Their analysis revealed a minor core microbiota, and a major variable fraction of the microbiota that changes with location, attributed to pollution and local anthropogenic disturbances [29]. Additionally, changes in their bacterial community were observed over time, with changes in composition linked to seasonal environmental changes [30]. The first studies examining the potential impact of heatwaves on microbiota assemblages of *L. sarmentosa* [31, 32] indicated changes in the composition of the associated microbiota and identified particular microbes as possible indicators of health status [31]. Overall, this adaptability to local and environmental conditions also suggests that *L. sarmentosa* can adapt to diverse conditions.

Visually healthy *L. sarmentosa* colonies can thrive in low-quality waters such as those in the seaport area, near Barcelona, typically characterized by freshwater input, high nutrient loading, hypoxia, and acidification. However, the microbiota signatures of these colonies remain unknown, limiting our understanding of the acclimation strategies that enable this species to persist in such highly stressed environments. Given the reported high densities of this species in disturbed areas [3], along with the growing interest in uncovering the underexplored biodiversity found in port ecosystems [33], it is essential to investigate the mechanisms that support this species’ resilience and adaptation in such urbanized habitats. Thus, the main objective of this study is to understand the acclimation strategies of *L. sarmentosa* by comparing the microbiota signatures in two contrasting environments: a marine protected area (MPA Cap de Creus) and a highly anthropogenically impacted site (Barcelona seaport). In order to provide a broader perspective on the environmental drivers of the *L. sarmentosa* microbiota, we further compared in parallel these data with a re-analysis of existing datasets from similarly contrasting Mediterranean sites (Cassis and La Spezia) [30]. This comparison will help us to understand what renders this species so resilient and may provide crucial insights for the advice of effective management of gorgonian coral communities. Furthermore, these insights serve as a baseline for predicting the long-term viability of Mediterranean marine biodiversity in the face of increasing coastal urbanization.

## Material and Methods

### Sampling Sites

**Fig. 1:**
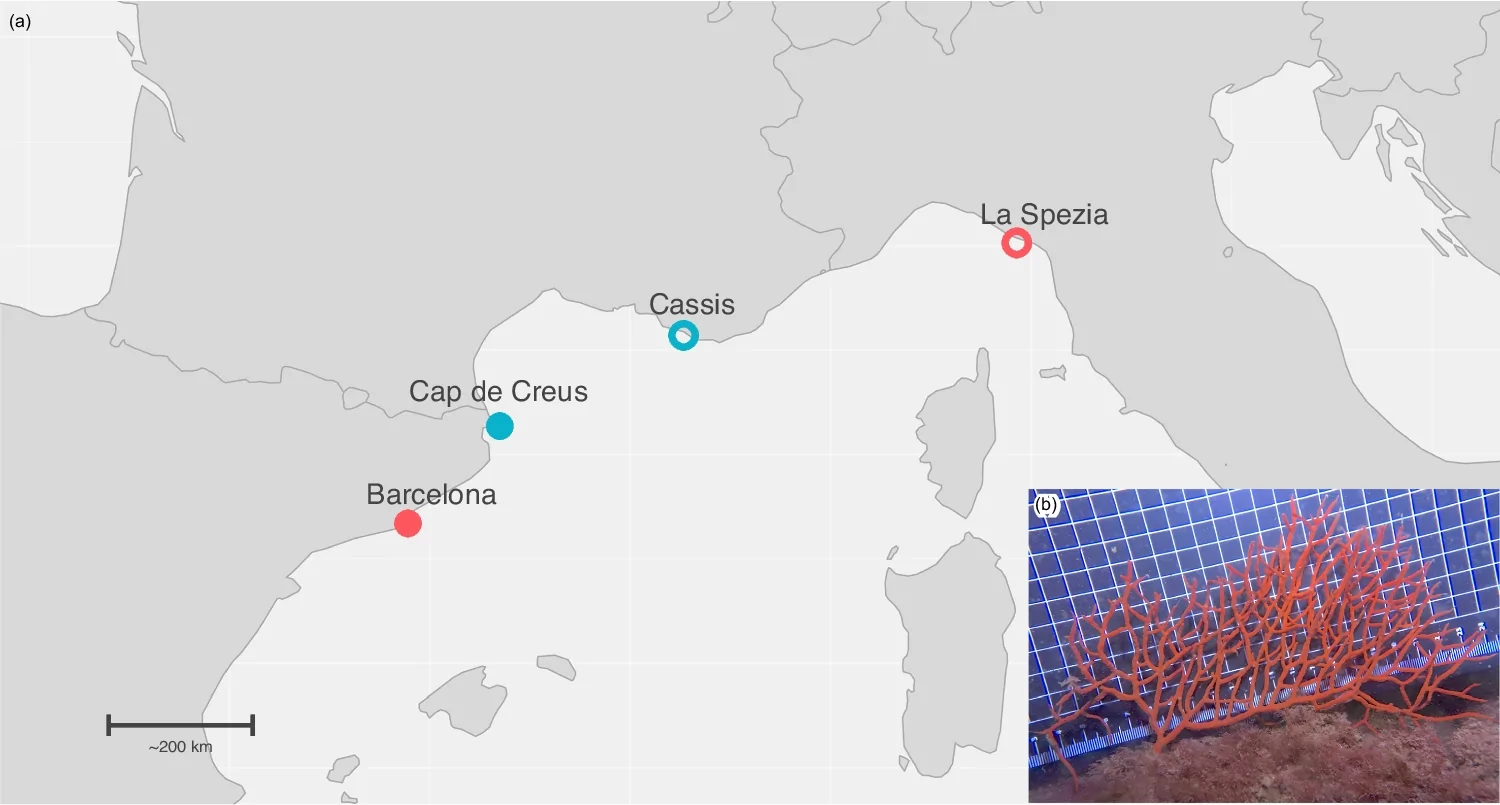
(**a**) Sampling sites in the Northwestern Mediterranean. Blue dots indicate less-impacted sites (MPA Cap de Creus and Cassis); red dots indicate highly urban-impacted sites (Barcelona seaport and La Spezia). (**b**) Representative *L. sarmentosa* colony from the Barcelona seaport. Empty circles denote sites for previous re-analyzed data. Photo: Oriol Deu

Samples were collected from two comparative sites: a seaport site in Barcelona (41°22′04.0″N, 2°11′29.0″E) and a site in the Marine Protected Area (MPA) of Cap de Creus (Western Mediterranean, 42º17′27.5’’N, 3º18′36.7’’E) (Fig. 1). At the seaport site, the sampling was conducted next to the submerged dike that functions as the external part of the harbour entrance, where monitoring of marine benthic ecosystems dominated by *L. sarmentosa* was done in the framework of the Gorgonia Barcelona Project [34]. This is an ambitious participatory scientific/citizen engagement project, aiming to improve the knowledge of Mediterranean corals and actively restore the Barcelona harbour seabed, dominated by this species. The seaport site is located near Barcelona’s main artificial beach (Barceloneta) and due to the several piers built near the area and the high rates of erosion, there are continuous inputs of river sediments, resulting in a seabed composed of fine particles. The area was given a “black flag” in the 2022 annual report of “Ecologistas en Acción”, primarily because of elevated pollution levels stemming from the intense maritime traffic linked to the nearby harbour of Barcelona [35].

Following sightings by Gori et al. (2011) [4], the samples of *L. sarmentosa* from the MPA site were collected from the soft bottoms near Illa Messina, in Cap de Creus (Fig. 1). The region’s hydrodynamic circulation pattern is primarily driven by the Northern current [36], influenced by intense and recurrent northern winds in the area [37]. *L. sarmentosa* individuals were found as scarce isolated colonies. Permits needed were obtained from the regional government to conduct studies on both sites, with a special permit in Cap de Creus provided by the Natural Park..

Colonies from van de Water et al. 2018 dataset were collected at two locations: La Spezia, Italy (44°01′ N, 09°50′ E; *n* = 5), and Cassis, France (43°12′ N, 05°28′ E; *n* = 4) (Fig. 1). The La Spezia locality is characterized by high turbidity, largely due to terrestrial runoff from the Magra River and impacted by the commercial maritime port and urban wastewater input from the city of La Spezia (Italy). This dataset also included colonies from an MPA near the Cassis locality (France) which experiences low turbidity, as it is not impacted by terrestrial runoff [30]. For consistency and comparability, only samples collected in March 2015, matching the month of our own sampling, were included in the re-analysis. Available abiotic factors for all four sites are summarized in Table 1.

**Table 1:** Environmental and abiotic characteristics across the four study sites (Cap de Creus, Barcelona seaport, Cassis, and La Spezia)

**Location**	**Cap de Creus**	**Barcelona seaport**	**Sources**
Coordinates (GSM coordinate system)	42º17′27.5’’N, 3º18′36.7’’E	41°22′04.0"N, 2°11′29.0"E	Measured in situ
Date	07/04/2022	09/04/2022	---
Depth (m)	20–22	19–21	Measured in situ
Temperature (ºC)	13.3	14	Measured in situ
Qualification of waters*	Good	Bad	Agencia Catalana de l’Aigua [112]
Hydrocarbon content*	---	Presence of benzene-based substances	Ecologistas en Acción. (2022)
pH	8.0–8.1	8.0–8.1	Hassoun et al. [113]
Turbidity	1 NTU	1–3 NTU	Copernicus Marine Service
Nutrients	nitrate 0.2 and 2 µM and phosphate 0.01–0.05 µM	nitrate 1–5 µM and phosphate 0.03–0.2 µM	Lazzari et al. [115], Wald et al. [116], Ibello et al. [114]
**Location**	**Cassis**	**La Spezia**	**Sources**
Coordinates (GSM coordinate system)	43° 12′ N, 05° 28′ E	44° 01′ N, 09° 50′ E	van de Water et al. 2018
Date	Mar-15	Mar-15	van de Water et al. 2018
Depth (m)	30–40	16–20	van de Water et al. 2018
Temperature (ºC)	13.25	13.31	Copernicus Marine Service
pH	8.2	8.17	Copernicus Marine Service
Turbidity	Low	High (total suspended solids concentration: 1.61–2.65 mg L-1)	Cites within van de Water et al. 2018

Parameters include depth, temperature, pH, turbidity, water qualification, hydrocarbon content, and nutrient concentrations (nitrate and phosphate). Data sources and specific references for each parameter are detailed in the final column of each respective section

### Data Collection, DNA Extraction and *16S rRNA* Gene Amplicon Sequencing

A total of 19 colonies of *L. sarmentosa* sea fans were sampled in the MPA Cap de Creus site (*n *= 6) and in the Barcelona seaport site (*n* = 13) in March 2022. All samples were collected between 19 and 22 m depth by scuba diving and within two consecutive days to minimize factors that could affect comparative analyses. The lower number of replicates at the MPA site compared to the seaport site was due to logistical constraints, including low colony density and challenging diving conditions (strong currents and low temperatures) typical of the NW Mediterranean in early spring. All sampled sea fans were visually healthy with no signs of necrosis or epiphytes. Sampled colonies were adults of approximately the same size to avoid potential age-related variations in microbial communities [38]. Sampling was standardized by collecting a 3 cm apical fragment from each sea fan to ensure tissue consistency and minimize colony impact. To prevent contamination, all gloves and tools used for sample collection and manipulation were either sterilized (using 70% ethanol followed by flame-sterilization) or discarded after single use. Each sample was gently rinsed with 100 ml of 0.22 µm-filtered seawater (FSW) to remove exogenous or transient microorganisms loosely associated with the coral tissue. Samples were stored in RNAlater (Thermo Fisher Scientific, Waltham, USA) and preserved at − 80 °C until subsequent DNA extraction.

Total DNA was extracted from the gorgonian samples using the DNeasy PowerSoil Kit (Qiagen, Hilden, Germany) according to the manufacturer’s instructions, with specific pre-processing steps. Briefly, coral tissue was macerated to a fine powder using a sterile mortar and pestle in the presence of liquid nitrogen. The resulting powder was then transferred to the kit's provided bead tubes for mechanical homogenization and subsequent chemical lysis. The variable V3-V4 region of the *16S rRNA* gene was sequenced in triplicate for each sample using the 341F/805R PCR primers [39, 40] and Illumina flow cell adapter sequences. PCR reactions were consolidated and cleaned with MinElute PCR Purification Kit (Qiagen) and quantified with a DS-11 FX + spectrophotometer (DeNovix, Wilmington, DE). DNA extraction, PCR reaction and sequencing of a kit blank was produced as a methodological control alongside regular DNA extractions. The resulting libraries were sequenced on an Illumina MiSeq platform using the 2 × 250 bp paired-end protocol. Raw sequencing reads are available at NCBI’s Sequence Read Archive under BioProject PRJNA1431356.

### Data Analyses

Adapters and primers were removed from raw sequencing reads with cutadapt v. 1.8.1 [41]. Amplicon libraries were analyzed in RStudio v. 1.1.456 with R v. 4.0.4. Quality filtering, error estimation, merging of reads, dereplication, removal of chimeras, and selection of amplicon sequence variants (ASVs) were performed with DADA2 v. 1.18.0 [42] using the filtering parameters: filterAndTrim{fnFs, filtFs,fnRs, truncLen = c(234,234), maxN = 0, maxEE = [c(2,2), truncQ = 2, rm.phix = TRUE, compress = TRUE, multithread = TRUE]}. Taxonomy was assigned to ASVs using the SILVA database v. 138 [43]. ASV and taxonomy tables were imported into phyloseq v. 1.34.0 [44] for analysis and visualization of microbial community structure. ASVs assigned as chloroplast, mitochondria, or eukaryote were removed from further analysis. ASVs labeled only as “Bacteria” were searched with BLASTn.

Alpha diversity metrics including the total number of observed ASVs, Shannon diversity, and Simpson diversity were calculated and rarefaction curves were generated to obtain a comprehensive description of the within-sample bacterial community. Differences in alpha-diversity metrics between sites were evaluated in R using either parametric or non-parametric tests, depending on the data distribution. Specifically, ANOVA was used to compare Shannon index and Observed ASV richness, while the Kruskal–Wallis test was applied to the Simpson index. Prior to multivariate analyses of beta diversity, we applied a Hellinger transformation on the ASV table and then calculated the Bray–Curtis dissimilarities between samples. Next, the Hellinger-transformed Bray–Curtis dissimilarity matrix was used (1) to visualize differences in bacterial community compositions between sites by applying a principal coordinate analysis (PCoA); (2) to perform a Permutational Analysis of Variance (PERMANOVA) to test for differences in bacterial community compositions (9999 permutations); and (3) to assess differences in multivariate dispersion based on the distance of samples to their respective group’s centroid using the betadisper function in vegan v. 2.5–7 [45]. Core microbiota of colonies from each site were analyzed (i.e., ASVs present in all samples).

Differential abundance (DA) analysis was performed using the ALDEx2 (version 1.34.0) [46] R package to account for the compositional nature of the microbiome data. The raw pathway counts were processed through a Dirichlet-multinomial model with 1,000 Monte Carlo iterations, followed by a Centered Log-Ratio (CLR) transformation. Statistical significance was assessed using Welch’s t-test on the CLR-transformed values. Results were interpreted based on the nominal p-value (*p* < 0.05) and the median difference between groups (diff.btw) to identify features enriched in either site comparison. To account for the unbalanced sampling design between seaport (*n* = 13) and MPA Cap de Creus (*n* = 6) and ensure biological relevance, taxa were also included if they exhibited a substantial effect size (|effect|> 1.0), which provides a more robust measure of difference than the nominal p-value alone in high-variance datasets.

The functional potential of the microbial communities was predicted using PICRUSt2 (v2.5.2) [47] based on the MetaCyc metabolic pathway database [48]. To account for the compositional nature of the microbiome data and the inherent uncertainty in functional predictions, differential abundance was assessed using ALDEx2 (v1.34.0). Significant differences between each site comparison were determined using Welch’s t-test (*p* < 0.05) on the CLR-transformed values, with the divergent pathways ranked by their median log-ratio difference (diff.btw).

To contextualize the microbiome signatures of *L. sarmentosa* across a broader geographical scale, we re-analyzed *16S rRNA* gene amplicon data from van de Water et al. (2018) [30]. While the original study targeted the V5–V6 hypervariable regions (primers 784F/1061R) [49], we processed the raw sequences through the identical bioinformatics pipeline and taxonomic database used for our V3–V4 data, as described above. Although the different primer sets necessitated separate processing, this re-analysis ensured that all taxonomic assignments were updated to current standards. This approach allowed for a robust comparison of site-specific signatures while controlling for both host species and seasonal variability (March).

The complete set of R scripts and metadata are available at https://github.com/elenaquintanilla-microbiome/Leptogorgia-sarmentosa

## Results

### Microbial Communities

**Fig. 2:**
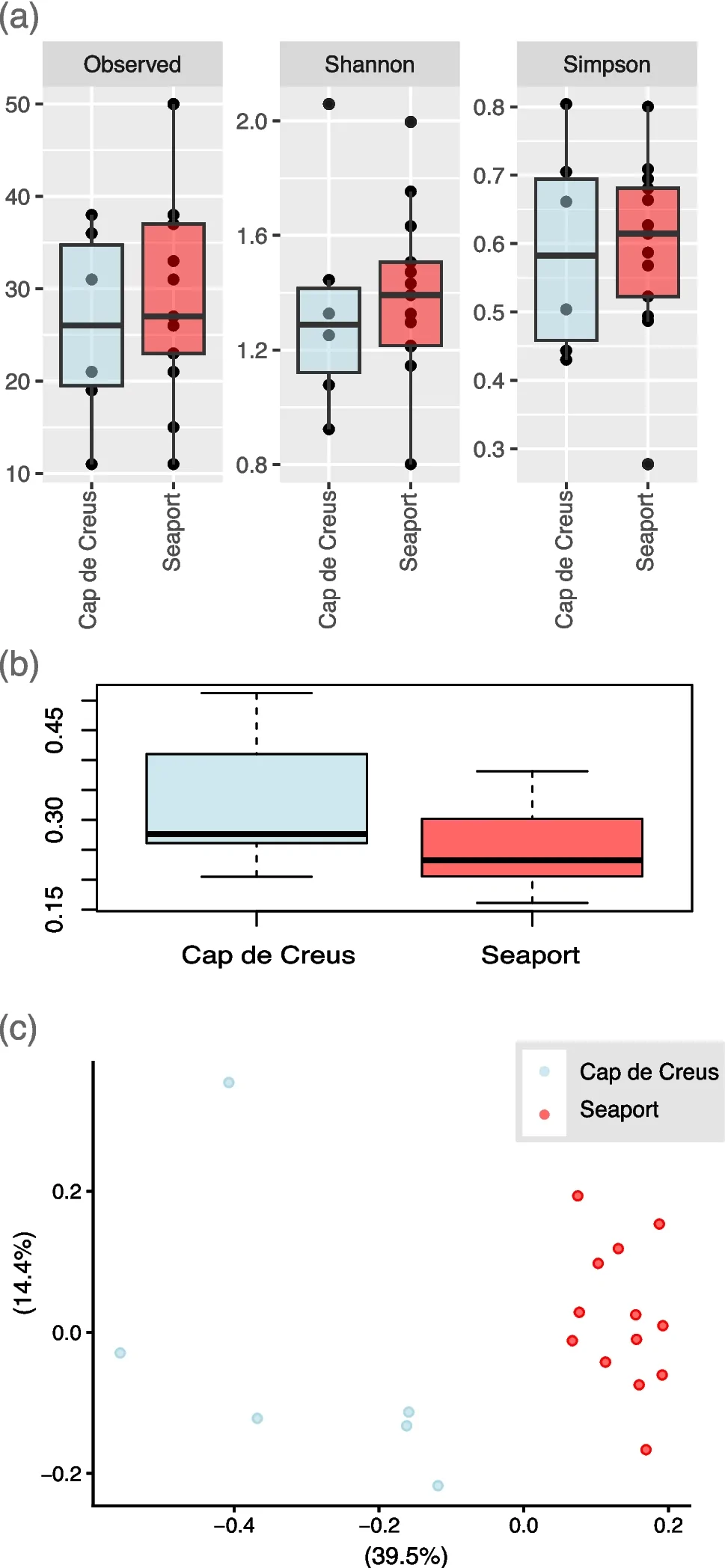
(**a**) Alpha diversity metrics (Observed richness and Simpson and Shannon index), (**b**) beta diversity dispersion based on Bray–Curtis distances; and (**c**) Principal Coordinates Analysis (PCoA) ordination of bacterial communities from the MPA Cap de Creus (in blue) and the Barcelona seaport (in red)

The final dataset (MPA vs seaport) consisted of a total of 19 gorgonian tissue samples. After filtering and merging, samples had an average of 27,405 reads per sample (10,462–44,711, see SM1 for DADA2 statistics). Rarefaction curves for all samples plateaued at 10,000 sequences, indicating a good representation of bacterial diversity (SM2 and SM3). We obtained a total of 356 ASVs (191 ASVs after removing singletons). Alpha-diversity metrics for *L. sarmentosa* colonies did not differ between sites in terms of richness (Observed ASVs, *p* = 0.535) or diversity (Shannon, *p* = 0.755; Simpson, *p* = 0.965) (Fig. 2A). Similarly, beta-diversity dispersions remained comparable between sites (*p* = 0.137) (Fig. 2B, SM4). Despite these similarities in diversity levels, Principal Coordinates Analysis (PCoA) and PERMANOVA revealed that microbial community compositions differed between the two studied locations (*p* = 0.001, R^2^ = 0.336; Fig. 2C, SM4).

**Fig. 3:**
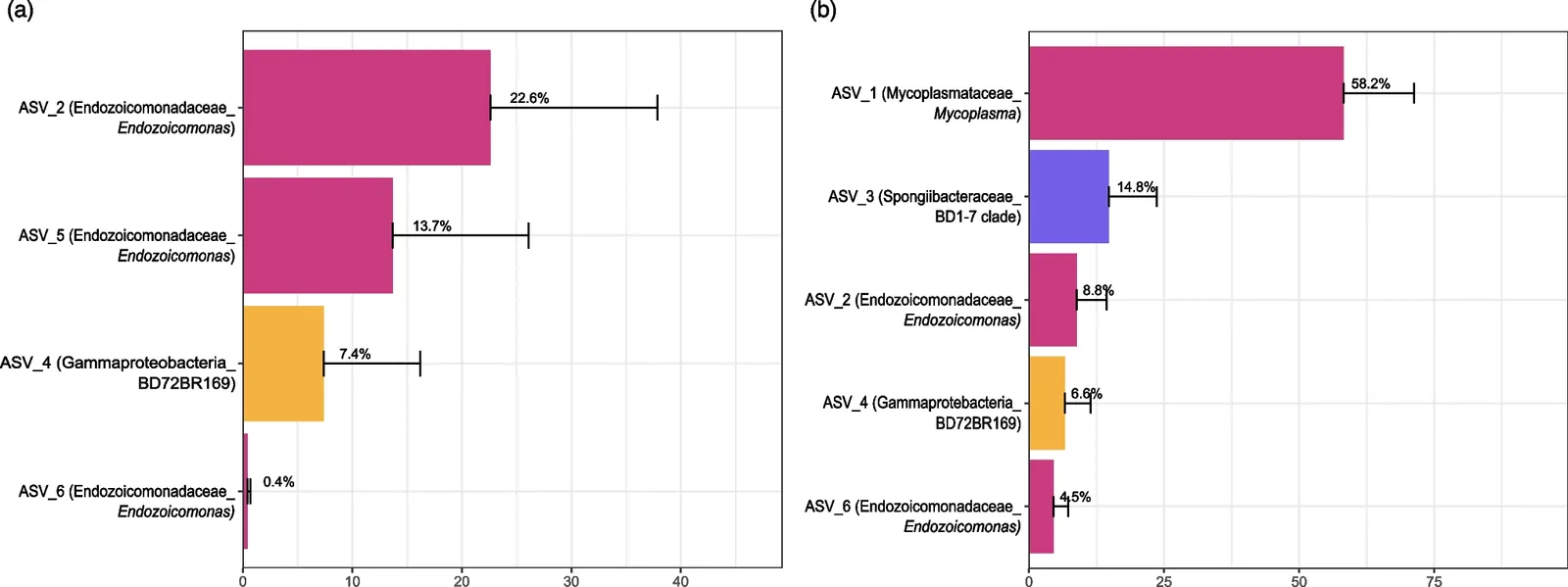
Relative abundance (% + SD) of ASVs constituting the *L. sarmentosa* core microbiota at (**a**) the MPA Cap de Creus and (**b**) the Barcelona seaport

The core microbiome of *L. sarmentosa* colonies at MPA Cap de Creus was characterized by four ASVs, showing a clear dominance of *Endozoicomonas* (ASVs 2, 5, and 6; 36.3% total abundance) and Gammaproteobacteria BD7-2-BR16-9 (ASV 4; 7.4%) (Fig. 3A). Conversely, the Barcelona seaport core microbiome consisted of five ASVs, with *Mycoplasma* (ASV 1; 58.2%), followed by *Spongiibacteraceae* BD1-7 (ASV 3; 14.8%), *Endozoicomonas* (ASV 2; ASV 6 13.3% total abundance), and *Gammaproteobacteria* BD72BR169 (ASV 4; 6.6%) (Fig. 3B).

**Fig. 4:**
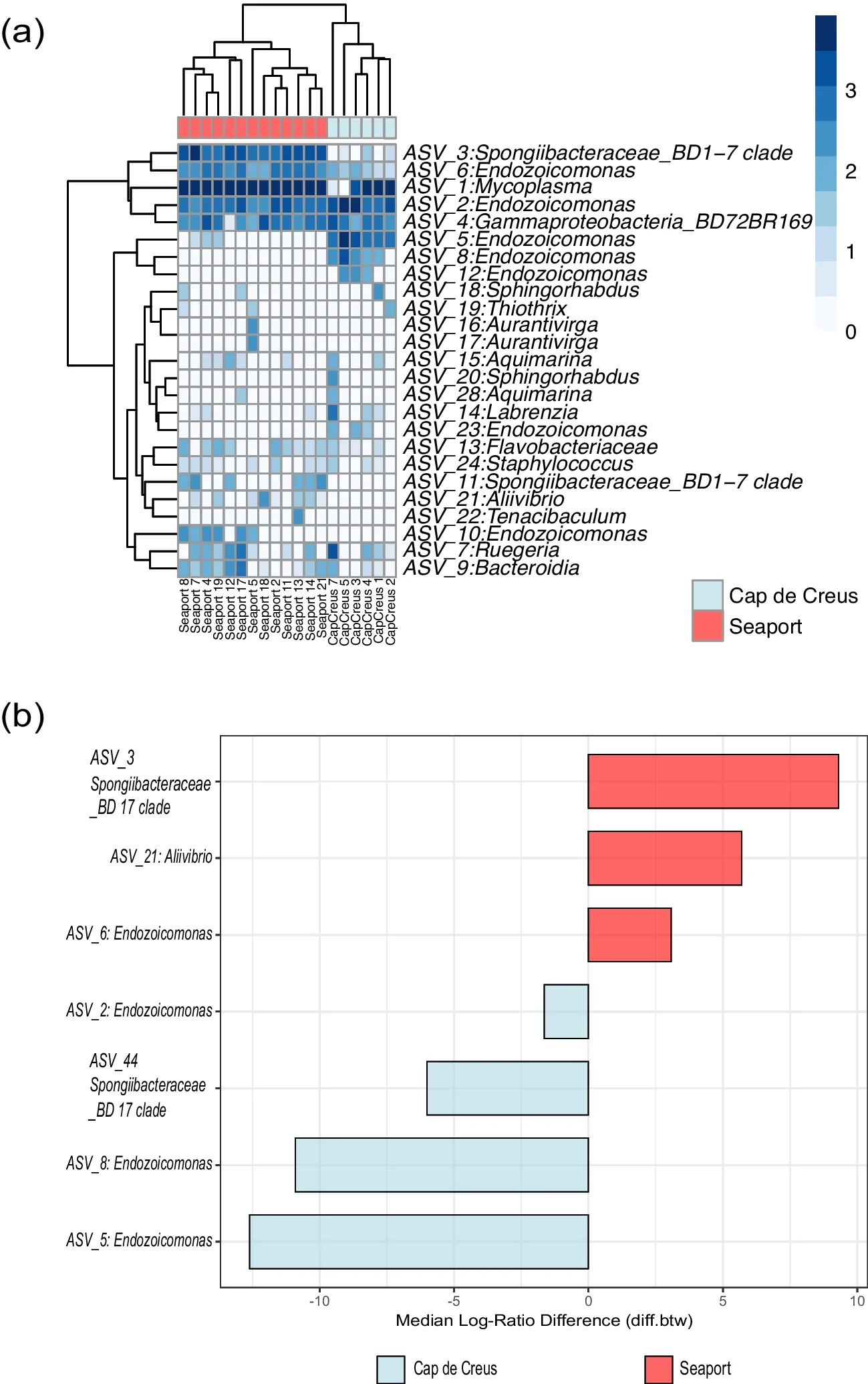
Taxonomic distribution of dominant and differential ASVs in *L. sarmentosa*. (**a**) log_10_-transformed relative abundances of the 25 most prevalent bacterial ASVs across colonies from the MPA Cap de Creus and the Barcelona seaport. (**b**) ALDEx2 differential abundance analysis highlighting significant ASVs enriched in each site (*p* < 0.05)

The divergent microbiome compositions of *L. sarmentosa* between the two sites were primarily driven by different ASVs. In general, the microbiota of colonies from MPA Cap de Creus were enriched in different *Endozoicomonas* ASVs (2, 5, 8) and *Spongiibacteraceae* BD1-7 (ASV 44). In contrast, the Barcelona seaport colonies showed higher abundances of members belonging to *Spongiibacteraceae* BD1-7 (ASV 3, 11), *Endozoicomonas* (ASVs 6) and *Aliivibrio* (ASV 21) (Fig. 4).

**Fig. 5:**
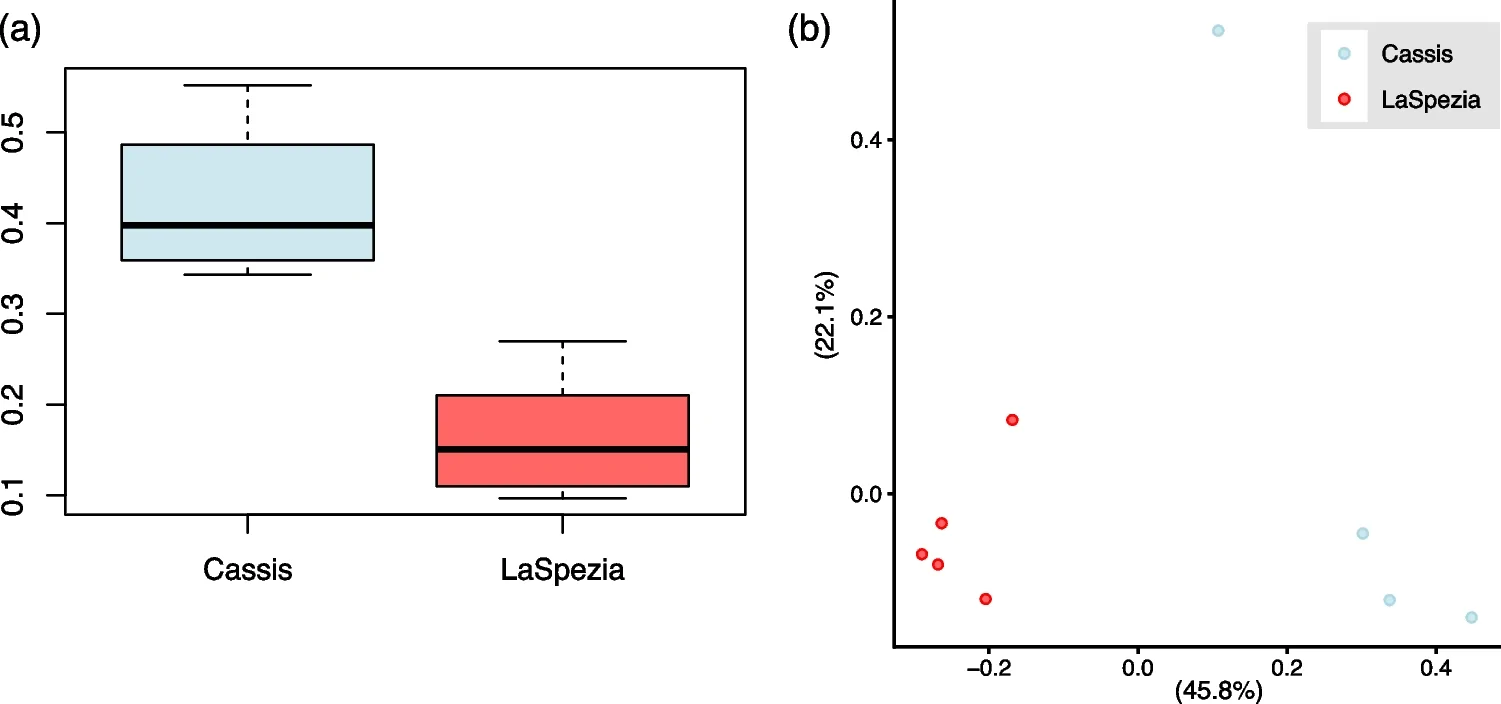
Microbial community structure of *L. sarmentosa* from re-analyzed datasets. (**a**) Beta diversity dispersion based on Bray–Curtis distances and (**b**) Principal Coordinates Analysis (PCoA) ordination of bacterial communities from the La Spezia (high-impact) and Cassis (low-impact) sites. Data represents a re-analysis of the original dataset from van de Water et al. (2018)

**Fig. 6:**
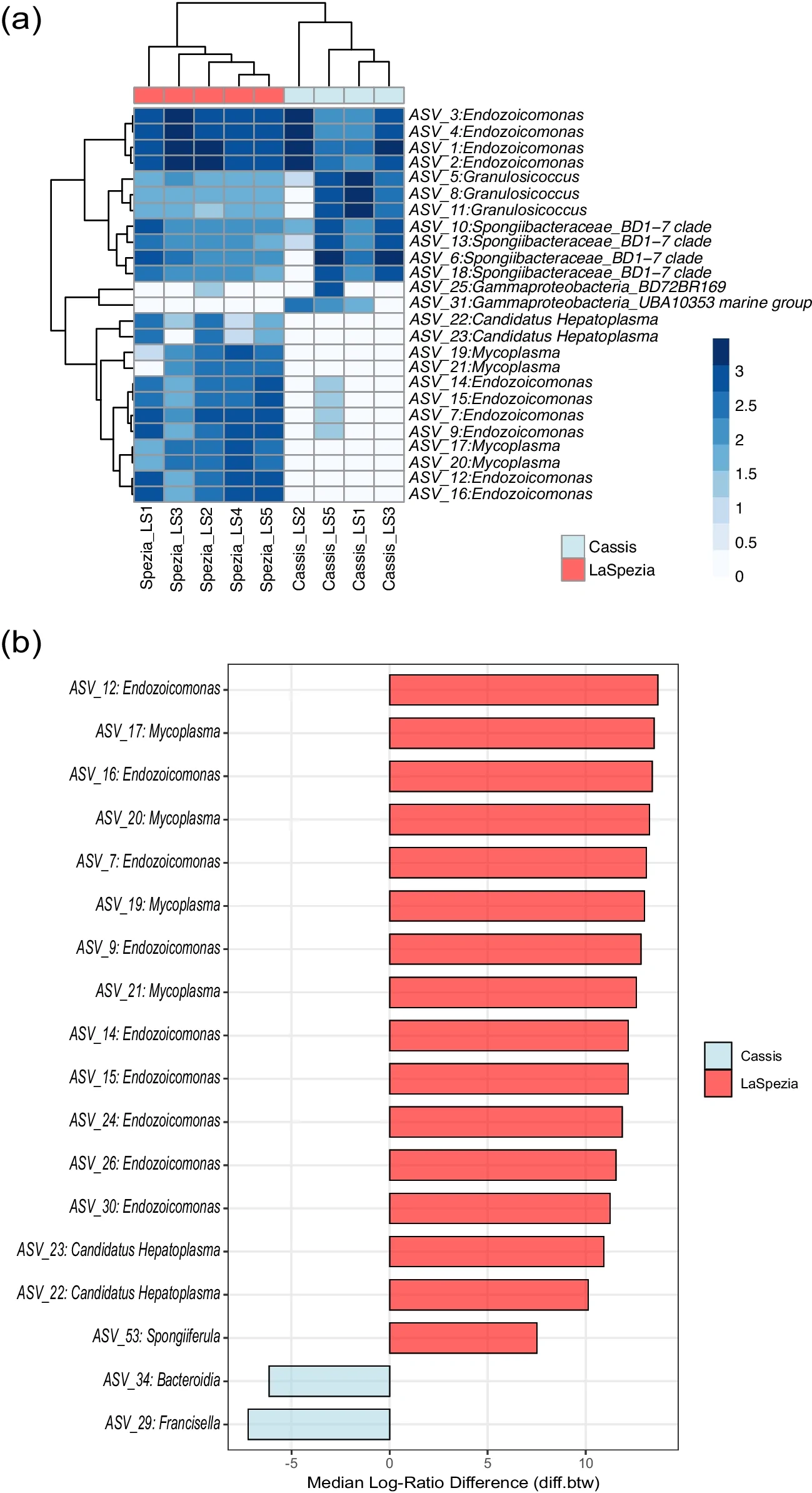
Taxonomic distribution of dominant and differential ASVs in *L. sarmentosa* from re-analyzed datasets. (**a**) log_10_-transformed relative abundances of the 25 most prevalent bacterial ASVs across colonies from La Spezia (high-impact) and Cassis (low-impact). (**b**) ALDEx2 differential abundance analysis highlighting significant ASVs enriched in each site (*p* < 0.05). Data are derived from the re-analysis of van de Water et al. (2018)

In the re-analysis of van de Water et al. (2018) [30] a total of 571 ASVs were obtained (371 ASVs after removing singletons. See SM5 for DADA2 statistics and SM6 for rarefaction curves). This dataset showed *L. sarmentosa* core microbiomes of colonies from La Spezia and Cassis (March 2015) dominated by *Endozoicomonas*, *Spongiibacteraceae BD1-7* clade, *Granulosicocaceae* and *Mycoplasma* (SM7 and SM8). Significant differences in the microbiota from both sites were observed in both beta-diversity dispersion (*p* = 0.001; Fig. 5A, SM5) and bacterial community composition (PERMANOVA: *p* = 0.01, R^2^ = 0.428; Fig. 5B, SM5). Generally, *L. sarmentosa* from the Cassis site harboured higher levels of strains of *Francisella* (ASV 29) and *Bacteroidia* (ASV 34). Conversely, a consortium of strains including *Endozoicomonas* (ASVs 7, 9, 14, 15, 16, 24, 26, 30), *Mycoplasma* (ASVs 17, 19–21) and *Ca Hepatoplasma* (ASVs 22, 23) was significantly higher abundant in corals from La Spezia (Fig. 6).

**Fig. 7:**
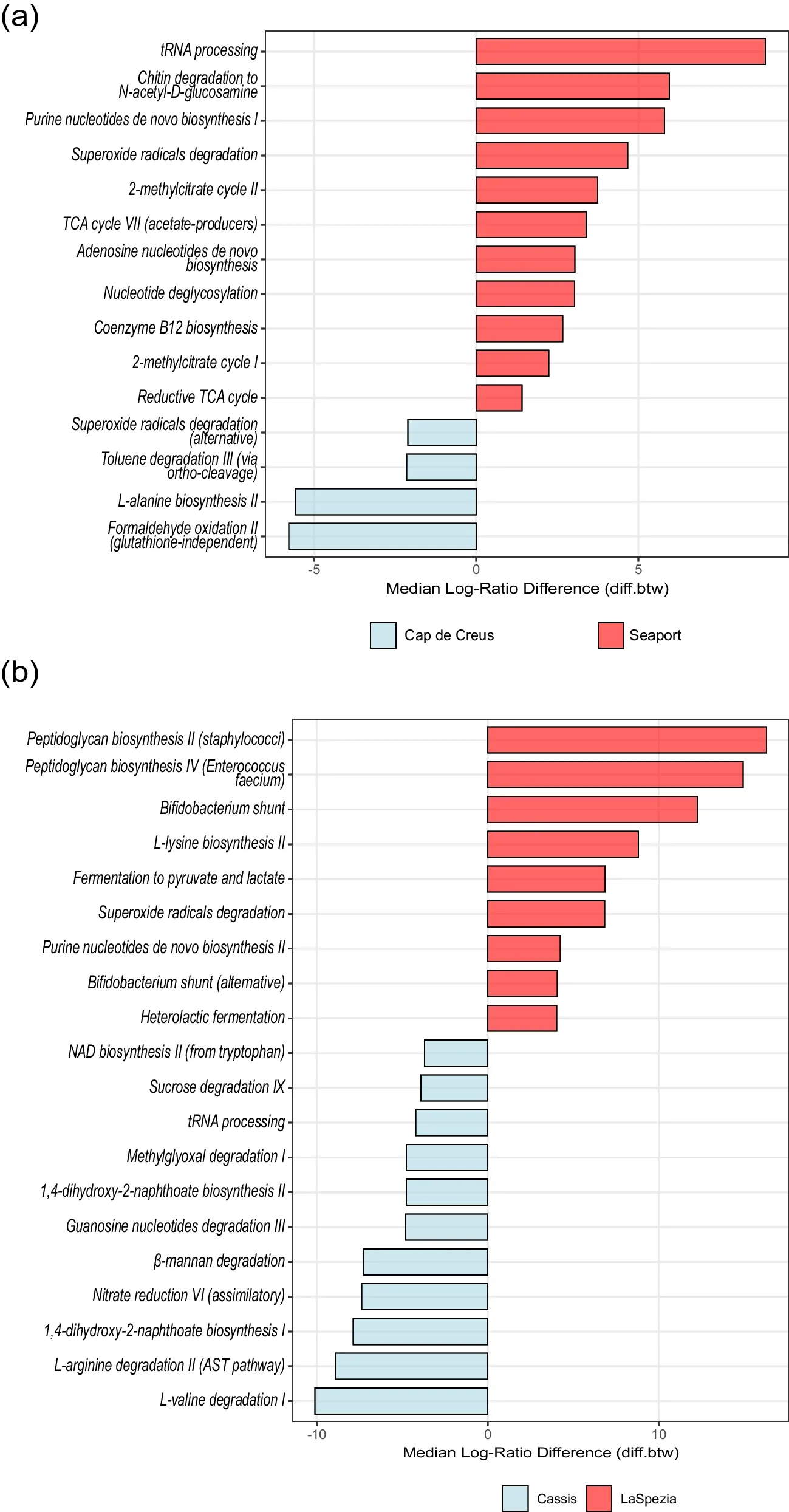
PICRUSt2 functional prediction and differential metabolic pathway abundance**.** ALDEx2 analysis highlighting significant shifts in microbial metabolic potential between distinct study sites based on Welch’s *p* < 0.05. (**a**) Enriched functional pathways in Barcelona seaport (red) versus MPA Cap de Creus (blue) samples. (**b**) Enriched functional pathways in La Spezia (red) versus Cassis (blue) samples

Overall, the functional predictive analysis performed with PICRUSt2 revealed distinct metabolic profiles between the highly impacted sites (La Spezia and Barcelona seaport) and their respective reference control sites (Cassis and MPA Cap de Creus). Specifically, in the comparison between seaport and MPA Cap de Creus, the microbiota of *L. sarmentosa* from the impacted environmentexhibited a significant enrichment in pathways including a marked increase in tRNA processing, *de novo* biosynthesis of purine and adenosine nucleotides, and nucleotide deglycosylation, 2-methylcitrate cycle (I and II) and the reductive TCA cycle, superoxide radicals degradation and chitin degradation pathways. In contrast, the microbiota of colonies from the MPA Cap de Creus exhibited a more specialized metabolic profile, characterized by the enrichment of formaldehyde oxidation II (glutathione-independent), L-alanine biosynthesis II and toluene degradation III (via ortho-cleavage) (Fig. 7A).

*L. sarmentosa* from La Spezia showed a pronounced enrichment in cell wall structural components, specifically peptidoglycan biosynthesis II and IV, alongside an increase in L-lysine biosynthesis II. Furthermore, a clear signal of specialized fermentation was observed in holobionts from this site, characterized by the enrichment of the *Bifidobacterium* shunt, heterolactic fermentation, and fermentation to pyruvate and lactate, together with an increase in superoxide radicals degradation. In contrast, the microbiota of *L. sarmentosa from* Cassis was characterized by a diverse array of degradative pathways and biosynthetic routes for secondary metabolites. This included a significant enrichment in the degradation of amino acids, such as L-valine degradation I, L-arginine degradation II (AST pathway), and NAD biosynthesis II. Additionally, it showed a higher abundance of pathways related to carbohydrate processing, such as beta-mannan degradation and sucrose degradation IX, as well as nitrate reduction VI (assimilatory) (Fig. 7B).

## Discussion

In this study, we identified distinct bacterial microbiome signatures in the holobionts of *Leptogorgia sarmentosa* from populations in the NW Mediterranean Sea naturally inhabiting environments with contrasting water quality: a marine protected area (the MPA Cap de Creus) and a highly anthropogenically impacted habitat (the seaport site near Barcelona city). By comparing these results with a re-analysis of data from van de Water et al. (2018) [30] (La Spezia and Cassis), we provide a broader perspective on the responses of *L. sarmentosa* microbiomes to highly impacted environments. Our findings reveal site-specific microbiota compositions, suggesting a high degree of microbiome flexibility, that allows this gorgonian to switch bacterial symbionts to potentially maintain the holobiont’s viability. Ultimately, our results highlight the remarkable capacity of *L. sarmentosa* to acclimate to highly disturbed local conditions through a stable yet plastic microbial association.

Significant differences in the bacterial community compositions of *L. sarmentosa* between the studied sites suggest that anthropogenic pressure may influence the coral microbiota. We observed a distinct compositional shift, characterized by a different repertoire of *Endozoicomonas* strains at each location. While *Endozoicomonas* remained a persistent member of the core microbiome in both sites, specific strains varied significantly; the relatively pristine MPA Cap de Creus colonies showed significantly higher abundances of ASVs 2, 5, 8, whereas Barcelona seaport colonies exhibited an enrichment of ASV 6. These findings align with the patterns identified in our re-analysis of *L. sarmentosa* microbiomes from van de Water et al. (2018) [30], where *Endozoicomonas* members dominated the core microbiome of colonies from La Spezia and Cassis, but a consortium of *Endozoicomonas* strains were significantly more abundant in the impacted environment of La Spezia. Members of the *Endozoicomonas* genus have been reported to be highly abundant in *L. sarmentosa* [30, 31] and are considered dominant endosymbionts within the core microbiomes of scleractinian and gorgonian corals[29, 50–53]. These bacteria are thought to contribute to coral health by producing antimicrobial compounds that inhibit pathogens [54, 55] and by participating in nutrient cycling processes [56, 57]. van de Water et al. (2017) [29]suggested that the diverse pool of *Endozoicomonas* species in the gorgonians’ core microbiome may share similar functions but may differ in their tolerance to environmental stressors. Additionally, alternate shifts in *Endozoicomonas* strains have also been suggested as an acclimation strategy of the coral holobiont to varying environmental conditions [58–60].

A core microbiota member assigned to the family *Spongiibacteraceae* BD1-7 (ASV 3) was significantly enriched in the microbiomes of *L. sarmentosa* colonies from the Barcelona seaport site. Members of this family have previously been found associated with gorgonians in deep-sea [61], mesophotic [51] and shallow waters [29, 30], as well as scleractinian corals [62–64], yet their role within the coral holobiont remains unknown. Notably, *Spongiibacteraceae* have been identified as hydrocarbon- and steroid-degrading bacteria [65, 66]. The heavy maritime traffic in the Barcelona seaport area may cause hydrocarbon pollution. These bacteria may aid the holobiont in removing these potentially damaging hydrocarbons and even convert these into a bioavailable carbon source for the coral, as has been previously hypothesized [67, 68]. Given that high concentrations of steroids are typically found in eutrophic marine environments, wastewater and urban runoff [69, 70], they may serve as a primary nutrient source for ASV 3 in the anthropogenically impacted Barcelona seaport environment. Furthermore, as several marine pathogens utilize steroids for infection and persistence [71], the enrichment of steroid-degrading bacteria could represent a holobiont protective strategy to neutralize potential virulence factors (*sensu* [66]). Several strains of this family were highly abundant in both Cassis and La Spezia sites from the van de Water et al. (2018) dataset. This suggests that *Spongiibacteraceae* may be a common associate of *L. sarmentosa* in the Mediterranean, with its prevalence potentially triggered by the terrestrial runoff and organic enrichment characteristic of urban coastal sites.

A single *Mycoplasma* strain dominated the core microbiome at the Barcelona seaport, where it accounted for 58.21% of the relative abundance, and remained highly abundant in most colonies from MPA Cap de Creus. These results are consistent with the parallel re-analysis of the van de Water (2018) dataset, which identified four *Mycoplasma* ASVs significantly more abundant in colonies from La Spezia urban site. While this genus is a frequent associate of both tropical and cold-water corals [72, 73] and other octocorals [29, 30, 51], its notable prevalence in these anthropogenically influenced sites is remarkable. Given that *Mycoplasma* has been suggested to participate in the adaptive response of the gorgonian holobiont during thermal anomalies[58], its abundance in impacted areas could reflect a potential contribution to maintaining holobiont homeostasis under environmental stress. Nevertheless, this potential link remains a hypothesis that needs further investigation.

Similarly, members of *Candidatus Hepatoplasma* were more abundant at the La Spezia site in the van de Water et al. (2018) dataset. Although the function of these bacteria is largely unknown, they have been found associated in cnidarians, gorgonians [53, 74], as well as in the gut microbiomes of crustaceans [75, 76]. It has been suggested that these bacteria function as gut symbionts potentially involved in the degradation of complex food sources[77], which could be particularly advantageous in nutrient-rich or runoff-impacted environments like La Spezia.

In contrast, members of the genus *Granulosicoccus* were more abundant in Cassis than in La Spezia, (representing 10.7% of the core microbiome in the less impacted area), which aligns with the original findings of van de Water et al. (2018). Although the specific ecological role of *Granulosicoccus* in gorgonians remains to be fully elucidated, this genus is a recognized constituent of bacterial communities on marine macrophytes and brown algae, such as *Fucus vesiculosus* [78, 79]. Furthermore, *Granulosicoccus* has been identified in bacterial biofilms that facilitate coral larval settlement [80]. Its higher prevalence in the less-impacted site (Cassis) compared to the urbanized location (La Spezia) leads to the hypothesis that *Granulosicoccus* might exhibit sensitivity to anthropogenic stressors or terrestrial runoff, pointing to its potential utility as an indicator of stable, oligotrophic environmental conditions.

The predictive functional profiling of the *Leptogorgia sarmentosa* microbiota revealed that the holobionts from the Barcelona seaport likely required microbes with a significant enrichment of key metabolic pathways compared to those from the pristine environment of Cap de Creus, in order to deal with chronic anthropogenic pressures (Table 1). The enrichment of the 2-methylcitrate cycle (cycles I and II) may reflect an active cellular detoxification mechanism. Benzene and other hydrocarbons are common in seaport waters and are partially degraded by marine hydrocarbonoclastic bacteria, often yielding propionate and other branched short-chain fatty acids as metabolic byproducts [81–83]. As free propionate is highly cytotoxic due to its inhibitory effect on central metabolism [84], the 2-methylcitrate cycle may serve as an important detoxifying pathway in this environment [85, 86]. This potential pollutant-driven metabolic processing also aligns with the enrichment of TCA cycle VII, which is characteristic of microorganisms living in acetate-rich environments [87]. The microbial breakdown of complex hydrocarbons typically yields substantial amounts of acetate alongside propionate [83, 88], and this overrepresentation may thus suggest that the seaport-associated microbiomes could exploit these chemical pollutants as an energetic subsidy. Concurrently, overrepresentation of *de novo* purine biosynthesis I, nucleotide deglycosylation and tRNA processing pathways suggests the microbes have an elevated requirement for genetic machinery maintenance, cellular turnover, and translational fidelity. Due to continuous exposure to xenobiotics and mutagenic pollutants in the seaport, microbes may needaccelerated DNA repair and cell proliferation, both of which depend on an immediate pool of purine nucleotides [89]. Further cellular preservation mechanisms are reflected by the enrichment of superoxide radicals degradation pathways, which suggest microbial mechanisms to neutralize destructive O_2_ radicals. This may be a consequence of the presence of heavy metals and benzene in the seaport area, and thus a need tomitigate oxidative damage and ultimately potentially sustaining the physiological homeostasis and survival of *L. sarmentosa* within the heavily anthropized environment [90, 91]. Furthermore, the enriched chitin degradation to N-acetyl-D-glucosamine may also reflect local euthrophic urbanized conditions. Through this chitinolytic pathway, some microbes within *L. sarmentosa* microbiomes could potentially benefit from recycling this particulate organic matter into an alternative carbon and nitrogen subsidy[92, 93].

A parallel trend was observed in the urbanization-impacted waters of La Spezia, where *L. sarmentosa* microbiomes were enriched in predicted functions like cell wall structural components (peptidoglycan and L-lysine biosynthesis) [94, 95] (and specialized fermentative pathways (such as the *Bifidobacterium* shunt and heterolactic fermentation), accompanied by a similar signal of superoxide radicals degradation. Taken together, these predicted profiles in both impacted areas suggest a common, yet independently observed, shift in the *L. sarmentosa holobiont* towards microbes able to neutralize potentially damaging compounds, with additional survival mechanisms, high cellular maintenance, and alternative energy harvesting under environmental degradation.

Overall, our findings suggest that *L. sarmentosa* colonies can restructure their microbiota in a highly impacted habitat through the selection of potentially more resistant microbes equipped with specific predicted functional pathways for pollutant detoxification, metabolic subsidy exploitation, and oxidative stress mitigation. The microbiota has been recognized to play an important role in the acclimation and resilience of corals under altered environmental conditions [58, 96]; but the ability of corals to dynamically restructure their microbiota may provide an alternate route to holobiont acclimation that facilitates rapid responses in the face of environmental change [97]. The microbiome flexibility hypothesis posits that this ability to respond and adapt to environmental change may take place in much shorter time than traditional organismal adaptation processes [28]. This microbiome flexibility may be related to the concept of functional redundancy, where different microbial taxa perform overlapping functions within the coral holobiont[28, 98]. By replacing sensitive microbes with more tolerant or functionally similar counterparts, the coral may preserve critical microbiome functions, thereby supporting holobiont acclimation and resilience under environmental pressures [98, 99]. Therefore, evidence found in this study suggests significant microbiome flexibility of *L. sarmentosa* by adjusting their microbiomes*,* potentially allowing its acclimation to highly anthropogenically impacted environments.

Furthermore, no significant differences were observed in the richness, diversity or dispersion of the microbiota of *L. sarmentosa* colonies between MPA Cap de Creus and Barcelona seaport. Stressed coral hosts typically exhibit elevated microbial diversity and richness, along with higher levels of dispersion, patterns characteristic of a disrupted microbiota and often referred to as the "Anna Karenina Principle" in microbial ecology [100–102]. However, our results reveal a noteworthy trend in which individuals from the MPA and Cassis exhibit a broader dispersion in compositional space compared to the tighter clustering observed in the Barcelona seaport and La Spezia. This observation stands in contrast to the Anna Karenina Principle; instead of environmental stressors increasing stochasticity, corals from the highly-impacted environments appear to harbor more homogenized microbial communities. Environmental stressors can destabilize microbiome function, leading to stochastic shifts in composition that facilitate the rise of opportunistic microbes and ultimately result in a dysbiotic state [100, 103, 104]. While such patterns have been documented in scleractinian corals exposed to sedimentation and sewage [105, 106] and in pollution-impacted gorgonians [107], our findings present a notable contrast. Despite inhabiting highly impacted sites, these *L. sarmentosa* populations appeared visually healthy and lacked the microbial hallmarks of disturbance-induced dysbiosis. Collectively, these observations suggest that *L. sarmentosa* remains in a stable physiological state despite environmental pressure, supporting the hypothesis that this species possesses a high capacity for acclimation through strategic microbiome reconfiguration.

These microbiome signatures coupled with known ecological traits place this gorgonian in the category of particularly resistant species. Colonies of *L. sarmentos*a are dwelling in environments with high nutrients and turbidity, on diverse bottom types, from gravel to soft bottoms and coralligenous substrates[1, 4, 6, 108], and even on concrete blocks in seaport areas with high anthropogenic impact [3]. Moreover, their life history traits also contribute to the resilience of this species, including high growth and feeding rates[1, 7, 109] shape and structural flexibility of the colonies [6, 110], and resistance to thermal stress [2, 3, 11]. Overall, these ecological features together with the microbiome signatures derived from this study, such as microbiome flexibility and the maintenance and acquisition of microbial functions important for holobiont health, suggest that *L. sarmentosa* is a gorgonian species well-adapted to differences in local environments, even under harsh conditions.

Our study shows that *L. sarmentosa* has the ability to resist challenging environmental conditions, supporting its potential as a suitable candidate for benthic conservation. These results also highlight the importance of implementing conservation measures and mitigate the anthropogenic impacts of harbor-related activities, to develop the potential of these areas as refuges for marine biodiversity [111]. Ultimately, this study sheds more light on the importance of integrating marine ecosystems, such as those dominated by *L. sarmentosa*, into urban management plans in Barcelona.

## Supplementary Information

Below is the link to the electronic supplementary material.

Supplementary file1 Quality filtering and read tracking stats across *Leptogorgia sarmentosa* samples (MPA Cap de Creus and Barcelona seaport) processed with the DADA2 pipeline. (TXT 1 KB)

Supplementary file2 Species rarefaction curves for *Leptogorgia sarmentosa* associated microbial communities sampled at MPA Cap de Creus. (PDF 60 KB)

Supplementary file3 Species rarefaction curves for *Leptogorgia sarmentosa* associated microbial communities sampled at Barcelona seaport. (PDF 99 KB)

Supplementary file4 Permutational multivariate analysis of variance (PERMANOVA) and permutation test for homogeneity of multivariate dispersions (PERMDISP) comparing microbial community structures across study sites. (XLSX 9 KB)

Supplementary file5 Quality filtering and read tracking stats across samples (Cassis and La Spezia) processed with the DADA2 pipeline. (TXT 1 KB)

Supplementary file6 Species rarefaction curves for *Leptogorgia sarmentosa* associated microbial communities sampled at Cassis and La Spezia. (PDF 72 KB)

Supplementary file7 ASVs comprising the core microbiota of *Leptogorgia sarmentosa* colonies sampled at La Spezia. (PDF 35 KB)

Supplementary file8 ASVs comprising the core microbiota of *Leptogorgia sarmentosa* colonies sampled at Cassis. (PDF 33 KB)

## Data Availability

Raw sequencing reads are available at NCBI’s Sequence Read Archive under BioProject PRJNA1431356. The complete set of R scripts and metadata are available at https://github.com/elenaquintanilla-microbiome/Leptogorgia-sarmentosa.
