# Supplementary material for: Bacterial Microbiota Signatures Suggest Acclimation of the Gorgonian Leptogorgia sarmentosa to Highly Impacted Environments in the Mediterranean Sea
Source: Microb Ecol. 2026 Jun 18;89:165. doi: 10.1007/s00248-026-02809-z (PMC13518739; doi:10.1007/s00248-026-02809-z)
Supplement: Supplementary file 1 [file 248_2026_2809_MOESM2_ESM.pdf]

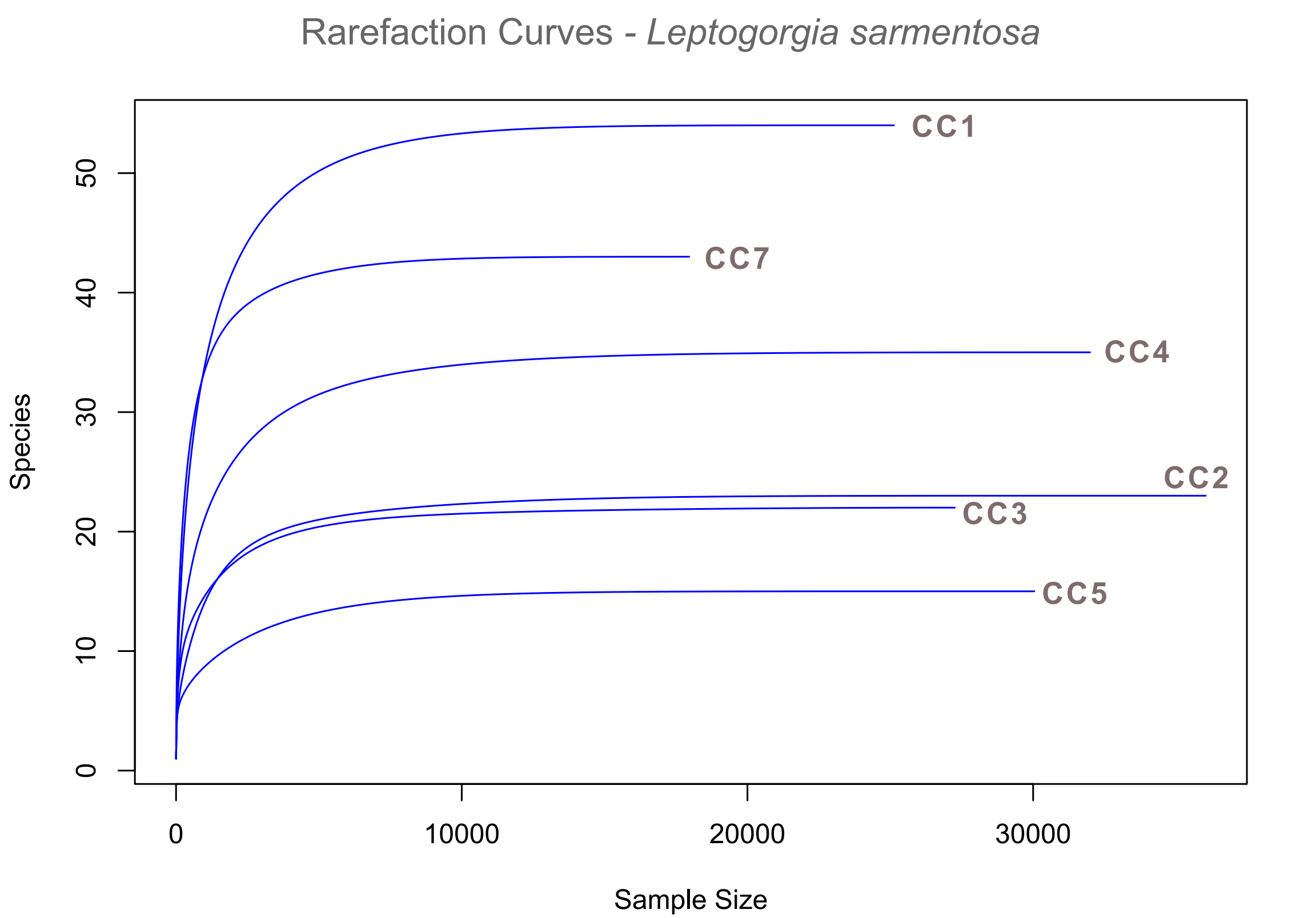
Rarefaction Curves - *Leptogorgia sarmentosa*
Species
Sample Size
0
10
20
30
40
50
0
10000
20000
30000
CC1
CC7
CC4
CC2
CC3
CC5
