# Supplementary material for: Bacterial Microbiota Signatures Suggest Acclimation of the Gorgonian Leptogorgia sarmentosa to Highly Impacted Environments in the Mediterranean Sea
Source: Microb Ecol. 2026 Jun 18;89:165. doi: 10.1007/s00248-026-02809-z (PMC13518739; doi:10.1007/s00248-026-02809-z)
Supplement: Supplementary file 3 [file 248_2026_2809_MOESM6_ESM.pdf]

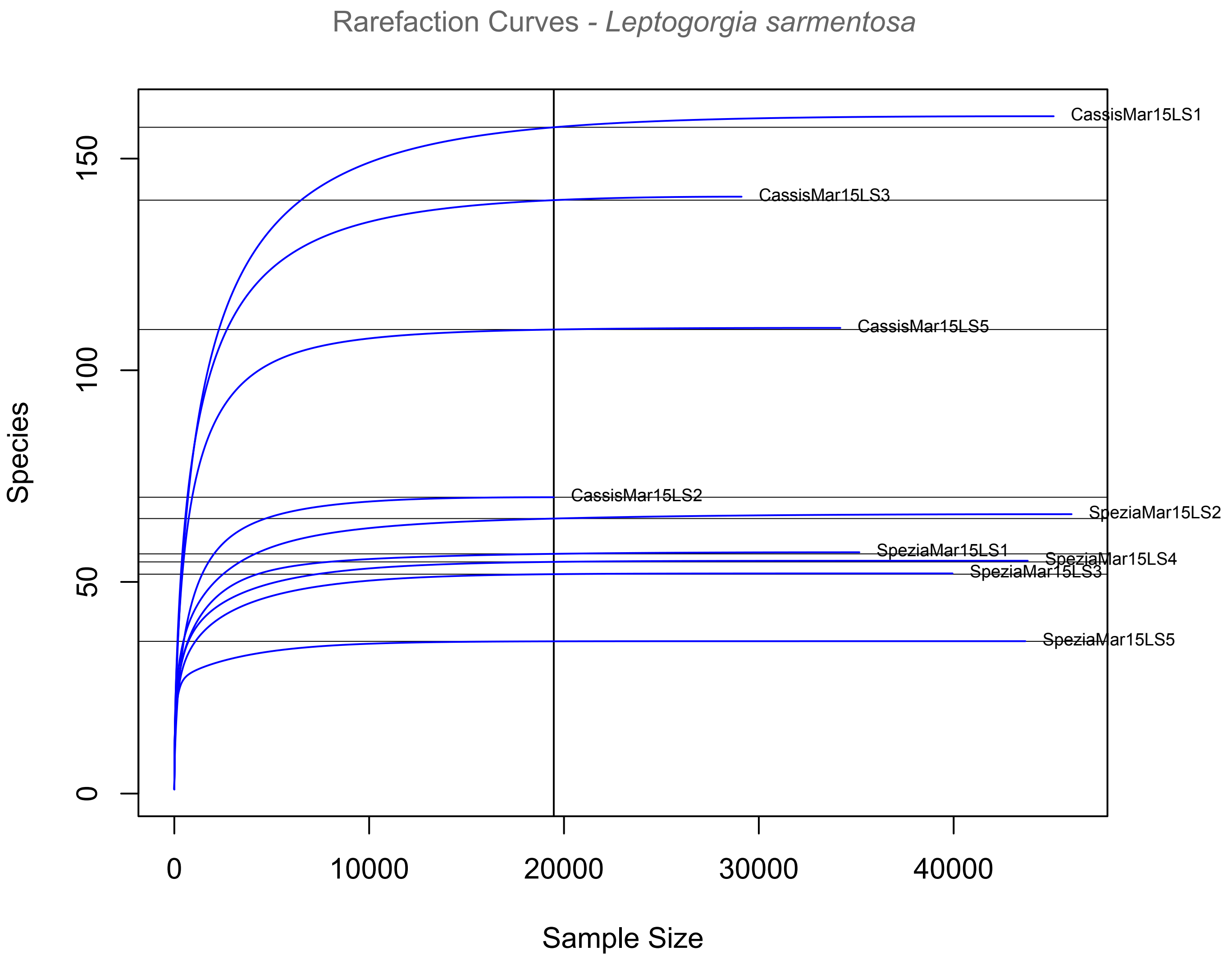

Rarefaction Curves - *Leptogorgia sarmentosa*
Species
Sample Size
0
50
100
150
0
10000
20000
30000
40000
CassisMar15LS1
CassisMar15LS3
CassisMar15LS5
CassisMar15LS2
SpeziaMar15LS2
SpeziaMar15LS1
SpeziaMar15LS4
SpeziaMar15LS3
SpeziaMar15LS5
