# Supplementary material for: Bacterial Microbiota Signatures Suggest Acclimation of the Gorgonian Leptogorgia sarmentosa to Highly Impacted Environments in the Mediterranean Sea
Source: Microb Ecol. 2026 Jun 18;89:165. doi: 10.1007/s00248-026-02809-z (PMC13518739; doi:10.1007/s00248-026-02809-z)
Supplement: Supplementary file 4 [file 248_2026_2809_MOESM7_ESM.pdf]

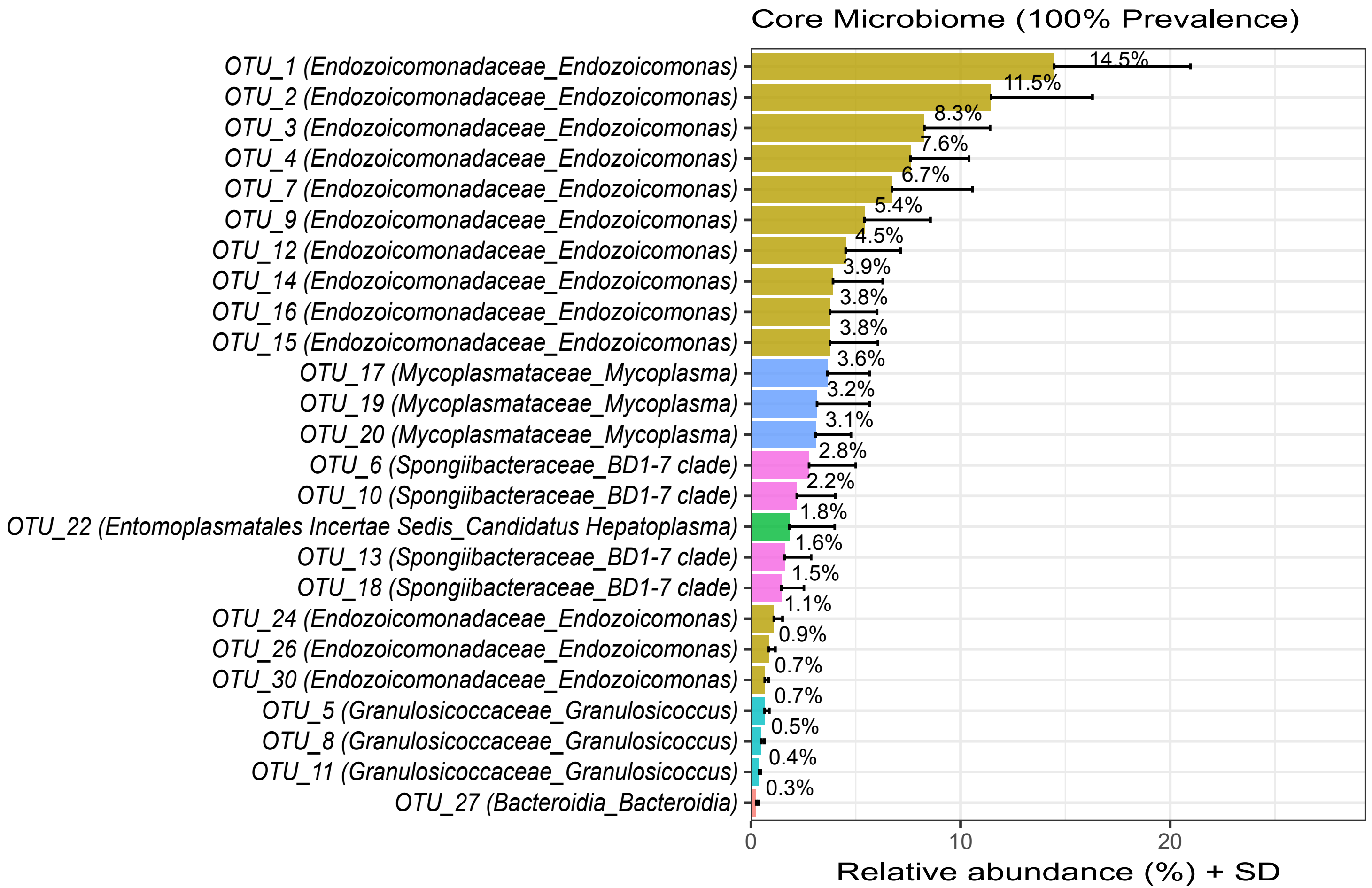
Core Microbiome (100% Prevalence)
OTU_1 (Endozoicomonadaceae_Endozoicomonas)
OTU_2 (Endozoicomonadaceae_Endozoicomonas)
OTU_3 (Endozoicomonadaceae_Endozoicomonas)
OTU_4 (Endozoicomonadaceae_Endozoicomonas)
OTU_7 (Endozoicomonadaceae_Endozoicomonas)
OTU_9 (Endozoicomonadaceae_Endozoicomonas)
OTU_12 (Endozoicomonadaceae_Endozoicomonas)
OTU_14 (Endozoicomonadaceae_Endozoicomonas)
OTU_16 (Endozoicomonadaceae_Endozoicomonas)
OTU_15 (Endozoicomonadaceae_Endozoicomonas)
OTU_17 (Mycoplasmataceae_Mycoplasma)
OTU_19 (Mycoplasmataceae_Mycoplasma)
OTU_20 (Mycoplasmataceae_Mycoplasma)
OTU_6 (Spongiibacteraceae_BD1-7 clade)
OTU_10 (Spongiibacteraceae_BD1-7 clade)
OTU_22 (Entomoplasmatales Incertae Sedis_Candidatus Hepatoplasma)
OTU_13 (Spongiibacteraceae_BD1-7 clade)
OTU_18 (Spongiibacteraceae_BD1-7 clade)
OTU_24 (Endozoicomonadaceae_Endozoicomonas)
OTU_26 (Endozoicomonadaceae_Endozoicomonas)
OTU_30 (Endozoicomonadaceae_Endozoicomonas)
OTU_5 (Granulosicoccaceae_Granulosicoccus)
OTU_8 (Granulosicoccaceae_Granulosicoccus)
OTU_11 (Granulosicoccaceae_Granulosicoccus)
OTU_27 (Bacteroidia_Bacteroidia)
14.5%
11.5%
8.3%
7.6%
6.7%
5.4%
4.5%
3.9%
3.8%
3.8%
3.6%
3.2%
3.1%
2.8%
2.2%
1.8%
1.6%
1.5%
1.1%
0.9%
0.7%
0.7%
0.5%
0.4%
0.3%
0
10
20
Relative abundance (%) + SD
