# Supplementary material for: Bacterial Microbiota Signatures Suggest Acclimation of the Gorgonian Leptogorgia sarmentosa to Highly Impacted Environments in the Mediterranean Sea
Source: Microb Ecol. 2026 Jun 18;89:165. doi: 10.1007/s00248-026-02809-z (PMC13518739; doi:10.1007/s00248-026-02809-z)
Supplement: Supplementary file 5 [file 248_2026_2809_MOESM8_ESM.pdf]

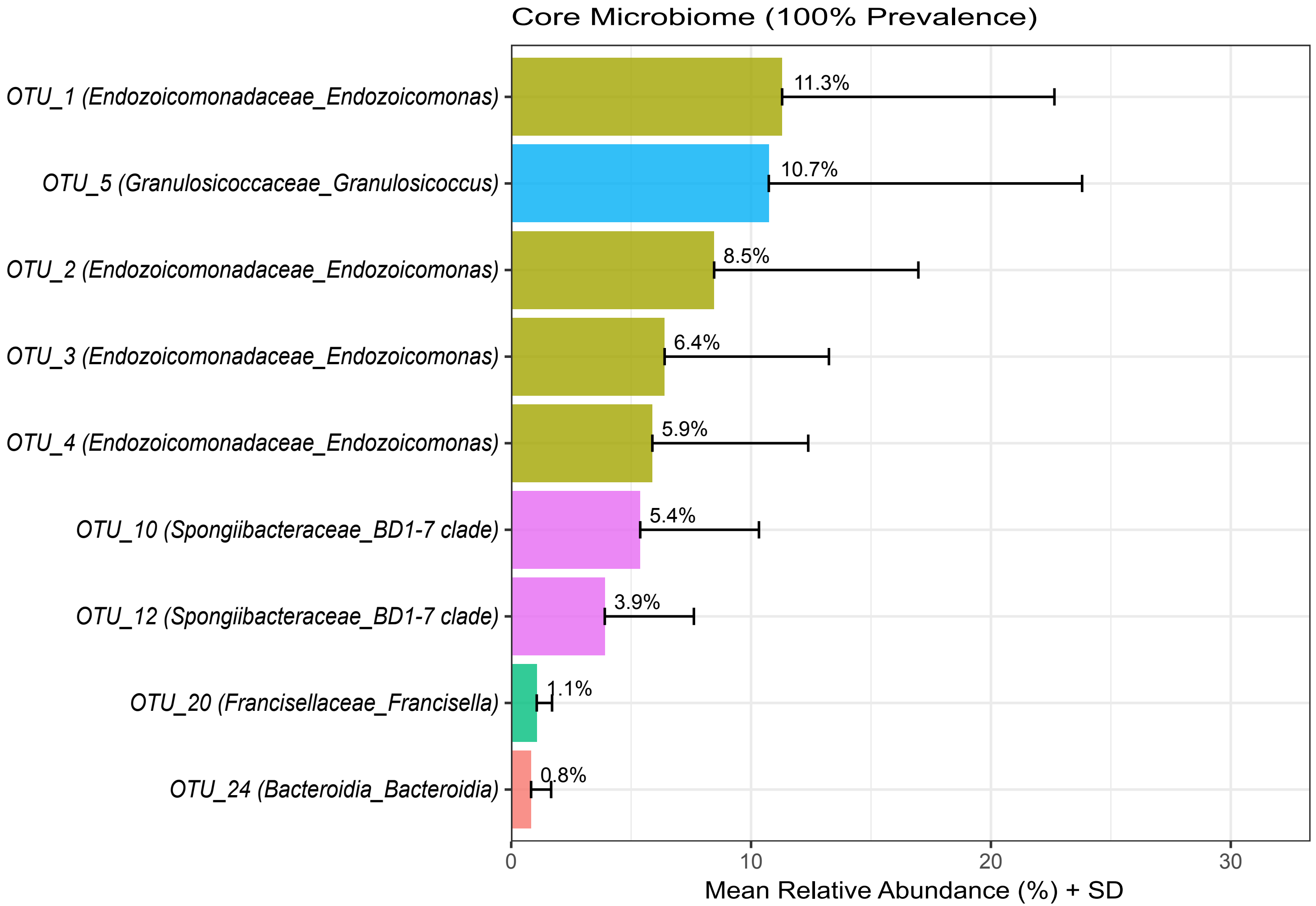
Core Microbiome (100% Prevalence)
OTU_1 (Endozoicomonadaceae_Endozoicomonas)
OTU_5 (Granulosicoccaceae_Granulosicoccus)
OTU_2 (Endozoicomonadaceae_Endozoicomonas)
OTU_3 (Endozoicomonadaceae_Endozoicomonas)
OTU_4 (Endozoicomonadaceae_Endozoicomonas)
OTU_10 (Spongiibacteraceae_BD1-7 clade)
OTU_12 (Spongiibacteraceae_BD1-7 clade)
OTU_20 (Francisellaceae_Francisella)
OTU_24 (Bacteroidia_Bacteroidia)
11.3%
10.7%
8.5%
6.4%
5.9%
5.4%
3.9%
1.1%
0.8%
0
10
20
30
Mean Relative Abundance (%) + SD
